# Cyclophosphamide induced early remission and was superior to rituximab in idiopathic membranous nephropathy patients with high anti-PLA2R antibody levels

**DOI:** 10.1186/s12882-023-03307-x

**Published:** 2023-09-22

**Authors:** Cheng Xue, Jian Wang, Jinyan Pan, Congdie Liang, Chenchen Zhou, Jun Wu, Shuwei Song, Linlin Cui, Liming Zhang, Yawei Liu, Bing Dai

**Affiliations:** 1 Division of Nephrology, Kidney Institute of CPLA, Shanghai Changzheng Hospital, Second Military Medical University (Navy Medical University), xuecheng@smmu.edu.cn, 415 Fengyang Road, Shanghai, 200000 China; 2 Department of Nephrology, No. 2 People’s Hospital of Fuyang City, Fuyang, 236000, Anhui Province China; 3 Department of Outpatient, Jinling Hospital, Nanjing, China; 4 Department of Nephrology, Zhabei Central Hospital of Jing’an District, Shanghai, China; 5 Outpatient Department, Yangpu Third Military Retreat, Shanghai, China; 6 Department of Internal Medicine, Changzheng Hospital, Second Military Medical University, Shanghai, China

**Keywords:** Rituximab, Membranous nephropathy, Cyclophosphamide, Meta-analysis, Treatment

## Abstract

Rituximab (RTX) and cyclophosphamide (CYC) based treatments are both recommended as first-line therapies in idiopathic membranous nephropathy (IMN) by KDIGO 2021 guideline. However, the efficacy of RTX vs. CYC-based treatments in IMN is still controversial. We performed this systemic review and meta-analysis registered in PROSPERO (CRD 42,022,355,717) by pooling data from randomized controlled trials or cohort studies in IMN patients using the EMBASE, PubMed, and Cochrane libraries (till Orc 1, 2022). The primary outcomes were the complete remission (CR) rate + partial remission (PR) rate. CR rate, immunologic response rate, relapse rate, and the risk of serious adverse events (SAE) were secondary outcomes. Eight studies involving 600 adult patients with IMN were included with a median follow-up duration of 12 to 60 months. RTX induced a similar overall remission rate compared with CYC (RR 0.88, 95% CI: 0.71, 1.09, *P* = 0.23). At the follow-up time of 6 months, RTX was associated with a lower CR + PR rate compared with CYC (RR 0.67, 95% CI: 0.52, 0.88, *P* = 0.003). Moreover, RTX might be less effective in inducing CR + PR than CYC treatment in IMN patients with high antiPLA2R antibody levels (RR 0.67, 95% CI: 0.48, 0.94, *P* = 0.02). The occurrences of CRs, relapse rates, immunologic response rates, and SAE were not significantly different between RTX and CYC, respectively. In conclusion, although the long-term efficacy and safety of CYC compared to RTX were comparable, CYC might respond faster and be more advantageous in IMN patients with high antiPLA2R antibody titers.

**Supplementary Information:**

The online version contains supplementary material available at 10.1186/s12882-023-03307-x.

## Introduction

The leading cause of nephrotic syndrome in adults is idiopathic membranous nephropathy (IMN). IMN is an immune-mediated disease characterized by subepithelial immune complex deposits and changes in the glomerular basement membrane^1^. Most patients with IMN have circulating autoantibodies against phospholipase A2 receptor (PLA2R) [1, 2], and 1–3% have antibodies against thrombospondin type-1 domain-containing 7 A (THSD7A) [3]. Some novel target antigens, such as neural epidermal growth factor-like 1 (NELL1), semaphorin 3B (Sema3B), protocadherin 7 (PCDH7), and high-temperature requirement A1 (HTRA1) have been identified in the remaining patients [4]. There is a strong correlation between antibody levels and progression risks in IMN associated with anti-PLA2R antibodies [5]. It is estimated that 5 to 30% of IMN patients with nephrotic syndrome experience spontaneous remission at five years, 15 to 30% have relapses, while 14 to 41% develop end-stage kidney disease over 15 years among those untreated patients with nephrotic syndrome [4].

Initially, patients with IMN receive supportive therapy; persistent nephrotic syndrome requires immunosuppressive therapy [6, 7]. IMN is mainly associated with B-cell dysfunction and immune complex deposition [4]. Most patients respond to alternating glucocorticoids and cyclophosphamide (CYC), but this regimen is associated with significant side effects, including hyperglycemia, infections, infertility, myelosuppression, and cancer [8, 9]. CYC decreases the production of nephrotoxic antibodies by profoundly but unselectively depleting B cells [10]. In recent years, rituximab (RTX), a monoclonal antibody that selectively depletes CD20 + B lymphocytes, shows promise in IMN treatment [11]. The Kidney Disease: Improving Global Outcomes (KDIGO) 2021 guidelines [12] recommend RTX or CYC combined glucocorticoids for six months, or calcineurin inhibitor (CNI) depending on the risk stratification. However, there is still no direct meta-analysis comparing the benefit and safety of RTX and CYC for IMN patients.

The STARMEN trial [13]found that alternating treatment with CYC + corticosteroids was superior to sequential treatment with RTX + tacrolimus (TAC) in IMN. However, the RI-CYCLO trial [14] found no significant difference in RTX vs. CYC in MN patients. Moreover, van den Brand et al. [15] compared two cohorts treated with either RTX or CYC and steroids. In the RTX-treated group, the partial remission rate was lower. Considering the comparative evidence is controversial, we conducted this systemic review and meta-analysis to explore the efficacy and safety of RTX vs. CYC based treatments in IMN patients.

## Methods

This meta-analysis was registered in PROSPERO (CRD42022355717) and followed the PRISMA guideline. The search for relevant studies was performed using the EMBASE, PubMed, and Cochrane libraries till Orc 1, 2022. We used ‘rituximab or CD20 Antibody or Rituxan’, ‘cyclophosphamide or Cytophosphane or CYC’, AND ‘membranous nephropathy or Membranous Glomerulopathy or Heymann Nephritis or Membranous Glomerulonephritis’ as the MESH or keywords. The search strategy was listed in Supplement Table 1. There were no language or publication time limitations. Publications that addressed rituximab and cyclophosphamide in IMN were further reviewed.

Publications that met the following inclusion criteria were selected: (1) randomized controlled trials (RCTs) or cohort studies; (2) enrolled adult patients with IMN; (3) RTX and CYC were administrated in the treatments with or without other immunosuppressive agents like glucocorticoids, calcineurin inhibitors, etc.; and (4) The follow-up time was more than 6 months, with at least one of the following endpoints: complete remission (CR) rate, partial remission (PR) rate, relapse rate, immunologic response (IR) rate, or adverse events. The exclusion criteria were: (1) ages < 18; (2) patients with secondary MN; (3) patients with another glomerulonephritis besides MN; (4) studies using other types of anti-CD20 antibodies; and (5) letters, abstracts, reviews, or animal studies; (6) no data available for analysis. Selection of studies was done by XC, ZC, WJ, PJ, and LC.

The following information was extracted from the included studies by XC and DB independently: first author, year, study design, settings, country, treatments, gender, ages, follow-up times, the number of patients in each group, estimated glomerular filtration rate (eGFR), baseline serum albumin, serum creatinine, proteinuria, RTX doses, anti–PLA2R antibody (antiPLA2Rab) positivity, numbers of CR, PR, and relapse. Any discrepancies between the two authors were solved by a discussion with a third author. CR was defined as a reduction of urinary protein: creatinine ratio (UPCR) from baseline to a value < 0.3 g/g plus stable eGFR; PR as a reduction of UPCR > 50% from baseline and a value < 3.5 g/g plus stable eGFR. Relapses were defined as a reappearance of proteinuria > 3.5 g/g. CR + PR rates were the primary outcomes. CR rate, immunologic response rate, relapse rate, and severe adverse events (SAE) were the secondary outcomes. Fenoglio et al.’s study had three arms with two of them using different amounts of RTX, we combined the RTX arms to perform the comparisons.

Assessment of the risk of bias in cohort studies was performed by two authors (XC and DB) independently. Cohort studies were evaluated using the Newcastle-Ottawa Scale (NOS) with the quality of selection, comparability, and exposure or outcome, while RCTs were evaluated by the Risk of bias tool (RoB2). The maximum score was nine points of NOS. The risk ratio (RR) and corresponding 95% confidence interval (CI) were used to compare the efficacy and safety of RTX and CYC in Review Manager 5.4. Statistical heterogeneity in the results was evaluated by *I*^*2*^ statistics. When *I*^*2*^ < 25%, there was low heterogeneity; when 25% < *I*^*2*^ < 75%, there was moderate heterogeneity; and when *I*^*2*^ > 75%, there was high heterogeneity. The random effects meta-analysis model was used in all results. The source of heterogeneity was explored by further subgroup analysis by different follow-up times, settings, drug combinations, and antiPLA2Rab cutoff levels (100 RU/ml). Sensitivity analysis was performed by excluding each study once at a time and changing models. Publication bias was investigated by the funnel plot, Begg’s test, and Egger’s test. A two-sided *P* value < 0.05 was considered statistically significant.

## Results

### Literature search

**Fig. 1:**
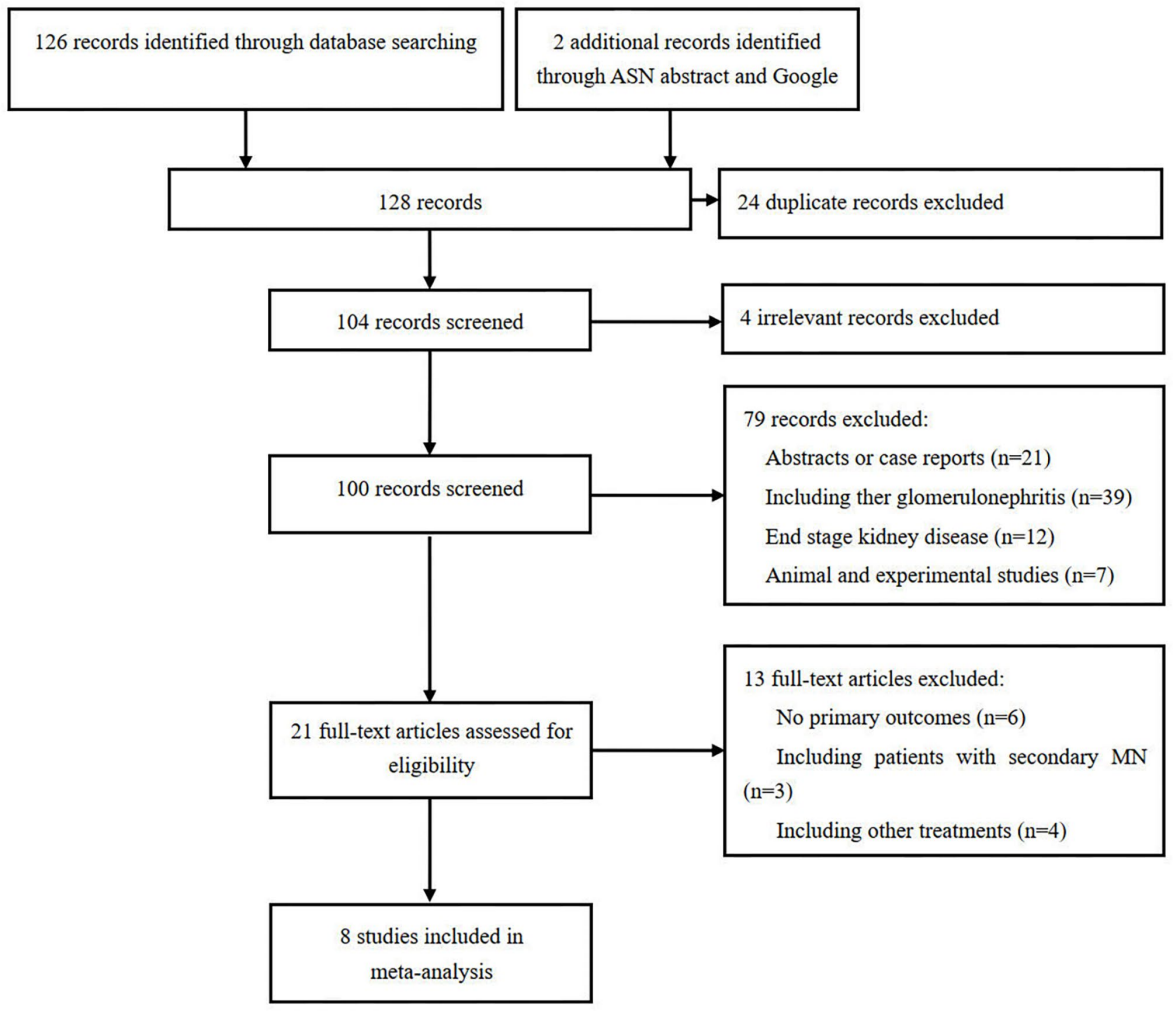
The flowchart of study identification, inclusion, and exclusion

The initial process of searching for relevant studies found 128 publications (Fig. 1). After the exclusion of 107 duplicates and irrelevant studies, 21 potentially eligible studies were further screened. Finally, 8 studies [13–18] involving 600 adult patients with IMN were included.

### Study characteristics

The baseline characteristics of the enrolled studies were shown in Table 1. There were 2 RCTs [13, 14], 2 prospective cohort studies [16, 18], and 4 retrospective cohort studies [15, 17, 19, 20]. Five studies [16–18] were performed in single centers and while the rest [13–15] were in multi-centers. Sample sizes of enrolled studies ranged from 36 to 203. The median follow-up time ranged from 12 to 60 months. The mean ages of included patients ranged from 48 to 67 years old. Six studies [13–15, 17, 18] were performed in Europe, and two studies [16, 20] in Asia. Most of the studies reported estimated glomerular filtration rate (eGFR): median range, 37 to 91 ml/min/1.73m^2^, serum creatinine levels: 0.9 to 1.7 mg/dl, and serum albumin levels: 2.0 to 2.6 g/dl. Six studies [14–17, 19, 20] compared RTX with CYC + prednisone, and two studies [13, 18] compared RTX + TAC with CYC + prednisone. AntiPLA2Rab levels were reported in six studies [13, 14, 16, 18, 20] with a median level of 58 to 259 RU/ml and used the same assay kit (Table 2). Median levels of proteinuria in the included studies were from 5.1 to 12.3 g/d. RTX dosages in different studies included 1 × 375 mg/m^2^ or 1 g [17], 4 × 375 mg/m^2^ (1-week interval), or 2 × 1 g (2 weeks interval).

**Table 1:** Characteristics of included studies

Author	Year	Country	Study design	Setting	Treatment	Sample size (n)	Run-in phase	Follow-up	Gender (M/F number)	Age (y)	PLA2Rabpositivity N (%)	PLA2Rab level (RU/ml)	Baseline proteinuria (g/d)	eGFR (ml/min/m^2^)	Scr (mg/dl)	Serum albumin (g/dl)	PLA2Rab assay company
Medrano [18]	2014	Spain	P	S	RTX + TAC	53	3 months	12 months	31/22	51.1 ± 14.2	53 (100)	239.8 ± 86	12.3 ± 3.6	91.6 ± 29.2	0.9 ± 0.4	2.2 ± 0.5	Euroimmune, Lubeck, Germany
					CYC + CS	26			18/8	51.8 ± 17.3	26 (100)	259 ± 89	11.9 ± 4.7	89.5 ± 36.3	1.0 ± 0.2	2.2 ± 0.4	
van den Brand [15]	2017	Netherlands and Italy	R	M	RTX	100	6 months	60 months	72/28	51.5 ± 15.9	NA	NA	6.4(4.4–8.9)	59.1 (26.6)	1.2 (1.0–1.7)	2.2 (0.7)	NA
					CYC + CS	103			76/27	55.3 ± 12.7	NA	NA	8.8 (5.7–11.7)	58.4 (22.8)	1.3 (1.0–1.6)	2.2 (0.7)	
Fenoglio [17]	2020	Italy	R	S	RTX low	14	6 months	24 months	9/5	64.4 ± 10.8	NA	NA	7.5 ± 4.8	68.7 ± 26.6	1.05 ± 0.34	2.5 ± 0.5	NA
					RTX standard	14			5/9	61.4 ± 11.5	NA	NA	5.1 ± 1.4	75.8 ± 29.8	1.06 ± 0.46	2.6 ± 0.6	
					CYC + CS	14			8/6	67.1 ± 17.5	NA	NA	8.3 ± 4.8	80.8 ± 29	1.3 ± 0.9	2.4 ± 0.5	
Fernandez-Juarez [13]	2020	Spain and Netherlands	RCT	M	RTX + TAC	43	6 months	24 months	31/12	55.2 ± 10.8	24/32 (75)	113 (61–151)	7.4 (6.7–11.6)	79.1 ± 25.5	1.0 ± 0.28	2.6 (2.0–2.9)	Euroimmune, Lubeck, Germany
					CYC + CS	43			24/19	56.2 ± 12.0	29/37 (78)	59 (37–150)	7.4 (4.8–11.3)	80.5 ± 21.6	1.0 ± 0.3	2.6 (2.3–2.9)	
Scolari [14]	2021	Italy and Switzerland	RCT	M	RTX	37	3 months	36 months	28/9	54 (14)	22 (73)	63 (52–87)	6 (4–10)	83 (24)	1 (0)	2 (1)	Euroimmune, Lubeck, Germany
‘					CYC + CS	37			25/12	55 (17)	19 (59)	58 (40–81)	6 (5–9)	86 (25)	1 (0)	2 (1)	
Ramachandran [16]	2021	India	P	S	RTX	13	NA	24 months	10/3	52 (36.5–59.5)	NA	194 (90–866)	7.9 (4.5–13.5)	37 (35–50.6)	1.6 (1.5–2)	2.4 (1.8–2.8)	Euroimmune, Lubeck, German
					CYC + CS	49			38/11	48 (41–55)	NA	126 (18–271)	5.2 (4–8)	42.5 (32–49)	1.7 (1.5–2.2)	2.5 (1.9–3.1)	
Hussain [19]	2022	United Kingdom	R	S	RTX or CYC	50	NA	12 months	30/20	53	18 (32)	NA	NA	NA	NA	NA	NA
Zhou [20]	2022	China	R	S	RTX	16	3 months	24 months	10/6	50 ± 10	16 (100)	210 ± 59	6.5 ± 4.2	72 ± 25	NA	NA	NA
					CYC + CS	20			11/9	52 ± 11	20 (100)	233 ± 62	5.6 ± 1.5	73 ± 33	NA	NA	

RTX, rituximab; CYC, cyclophosphamide; CS, cyclical steroid; R, retrospective cohort study; P, prospective cohort study; S, single-center; M, multi-center; n, number; M, male; F, female; y, year; eGFR, estimated glomerular filtration rate; Scr, serum creatinine; NA, not available

**Table 2:** Efficacy and severe adverse events in included studies

Author	Year	Treatment	Sample size (N)	CR + PR, N (%)	CR, N (%)	NR, N (%)	Relapse N (%)	SAE Patients, N (%)	Immunological response, N (%)	Funding (N)	RTX dose
Medrano [18]	2014	RTX + TAC	53	49 (92.5)	28 (57)	4 (7.5)	0 (0)	NA	77% at 12 months	None	RTX 1 g on days 1 and 15 or 4 weekly doses of 375 mg/m^2^
		CYC + CS	26	19 (73)	6 (32)	7 (27)	0 (0)	NA	58% at 12 months		
van den Brand [15]	2017	RTX	100	64 (64)	26 (26)	NA	NA	9 (9)	NA	3^a^	RTX 4 weekly doses of 375 mg/ m^2^
		CYC + CS	103	89 (86.4)	34 (33)	NA	NA	30 (29.1)	NA		
Fenoglio [17]	2020	RTX low	14	13 (92.8)	12 (85.7)	1 (8)	1 (8)	1 (8)	93% at 3–6 months	None	1 dose of RTX 375 mg/m^2^
		RTX standard	14	13 (92.8)	13 (92.8)	1 (8)	0 (0)	2 (15)	93% at 3–6 months		RTX 4 weekly doses of 375 mg/ m^2^
		CYC + CS	14	12 (85.7)	12 (85.7)	2 (17)	1 (8)	3 (25)	NA		
Fernandez-Juarez	2020	RTX + TAC	43	25 (58)	11 (26)	NA	3 (12)	6 (14)	45%, 70%, 79%, 83% at 3, 6, 12, and 18 months	10 ^b^	RTX 1 g
[13]		CYC + CS	43	36 (84)	26 (60)	NA	1 (2)	8 (19)	77%, 92%, 88%, 88% at 3, 6, 12, and 18 months		
Scolari [14]	2021	RTX	37	17/20 (85)	6/20 (30)	NA	3 (13)	7 (19)	63%, 62%, and 91% at 6, 12, and 18 months	None	RTX 1 g on days 1 and 15
		CYC + CS	37	16/22 (73)	7/22 (32)	NA	6 (22)	5 (14)	50%, 56%, and 73% at 6, 12, and 18 months		
Ramachandran [16]	2021	RTX	13	5 (38.5)	NA	NA	NA	5 (38.5)	NA	2^c^	RTX 4 weekly doses of 375 mg/ m^2^
		CYC + CS	49	24 (49)	NA	NA	NA	24 (49)	NA		
Hussain [19]	2022	RTX	25	10 (24)	4 (17)	NA	NA	NA	NA	None	NA
		CYC	25	19 (57)	5 (20)	NA	NA	NA	NA		NA
Zhou [20]	2022	RTX	16	10 (62.5)	NA	NA	NA	NA	NA	None	RTX 1 g
		CYC + CS	20	16 (80)	NA	NA	NA	NA	NA		

RTX, rituximab; CYC, cyclophosphamide; CS, cyclical steroid; CR, complete remission; PR, partial remission; NR, no response; SAE, severe adverse events; NA, not available. Immunologic response was defined by a level of antiPLA2Rab < 20 RU/ml. ^a^ European Union Seventh Framework Programme FP7/2007–2013 grant 305,608: European Consortium for High-Throughput Research in Rare Kidney Diseases. Dutch Kidney Foundation grants DKF14OKG07 and KJPB11.021. ^b^ Instituto de Salud Carlos III/Fondo Europeo de Desarrollo Regional (ISCIII/FEDER) grants PI13/02495 and ICI14/00350, Red de Investigación Renal (RedInRen) (RD12/0021/0029), ERA-EDTA, Fundación Renal Iñigo Álvarez de Toledo (FRIAT), Fundación para laInvestigación Biomédica Hospital 12 de Octubre (iþ12), Centre National de la Recherche Scientifique, Fondation Maladies Rares (LAM-RD_20170304), National Research Agency (ANR, grants MNaims ANR-17-CE17-0012-01), “Investments for the Future” Laboratory of Excellence SIGNALIFE, a network for innovation on signal transduction pathways in life sciences (ANR-11-LABX-0028-01), Initiative of Excellence (IDEX; UCAJedi ANR-15-IDEX-01), Fondation pour la Recherche Médicale (FRM, ING20140129210, DEQ20180339193, and FDT201805005509. ^C^ Indian Council of Medical Research (No. 5/4/7 − 5/14/NCD-II) and PGIMER-intramural fund

### Qualities of included studies

Quality ratings of included cohort studies [15–20] were listed in Supplement Table 2. Four studies [13–18] scored ≥ 7 points, while two studies [19, 20] scored 5 points with low quality. RoB2 showed that two RCTs [13, 14] were both open-labeled, and the risks of allocation concealment and blinding of participants were high (Supplement Fig. 1).

### Complete remission and partial remission rate

**Fig. 2:**
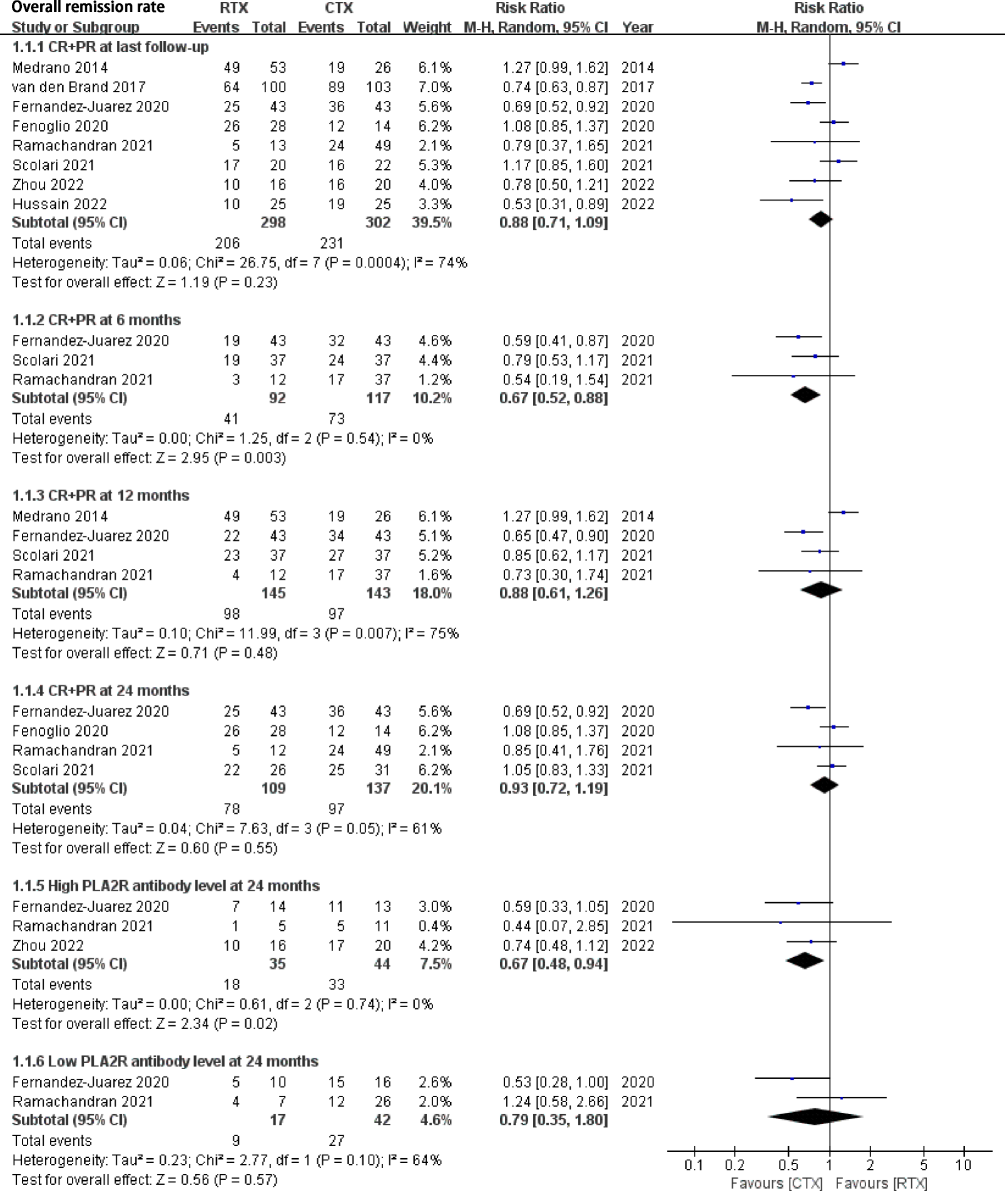
Comparison of complete and partial remission rate between rituximab and cyclophosphamide groups in IMN patients CR, complete remission; PR, partial remission

All included studies [13–18] addressed the rate of CR + PR. RTX treatment was associated with a similar probability of CR + PR rate compared with the CYC group at the last follow-up (RR 0.88, 95% CI: 0.71, 1.09, *P* = 0.23, Heterogeneity *I*^*2*^ = 74%, Fig. 2). Publication bias was not significant (Begg’s test: *P* = 0.707, and Egger’s test: *P* = 0.647, Supplement Fig. 2). Sensitivity analysis found stable results (Supplement Fig. 2).

At the follow-up time of 6 months, RTX was associated with a lower CR + PR rate compared with CYC (RR 0.67, 95% CI: 0.52, 0.88, *P* = 0.003). However, at the follow-up time of 12 months and 24 months (Fig. 2), there were no statistical significances of CR + PR rate between RTX and CYC (RR 0.88, 95% CI: 0.61, 1.26, *P* = 0.48; RR 0.93, 95% CI: 0.72, 1.19, *P* = 0.55, respectively).

Subgroup analysis by antiPLA2Rab levels also found different results. RTX was associated with a lower risk of CR + PR compared with CYC in patients with relatively high antiPLA2Rab levels (3 studies, RR 0.67, 95% CI: 0.48, 0.94, *P* = 0.02) but not in studies with lower antiPLA2Rab levels (2 studies, RR 0.79, 95% CI: 0.35, 1.80, *P* = 0.57). Subgroup analysis by different clinical settings and treatment combinations did not find significant differences in CR + PR rate (Supplement Fig. 3). Moreover, a subgroup analysis by the dosing of Rituximab (low: 1 g vs. standard dosing: RTX 1 g on days 1 and 15 or four weekly doses of 375 mg/m^2^). The results did not find statistically significant differences between low and standard dosing of RTX vs. CYC on CR + PR rate in IMN (Supplement Fig. 3).

**Fig. 3:**
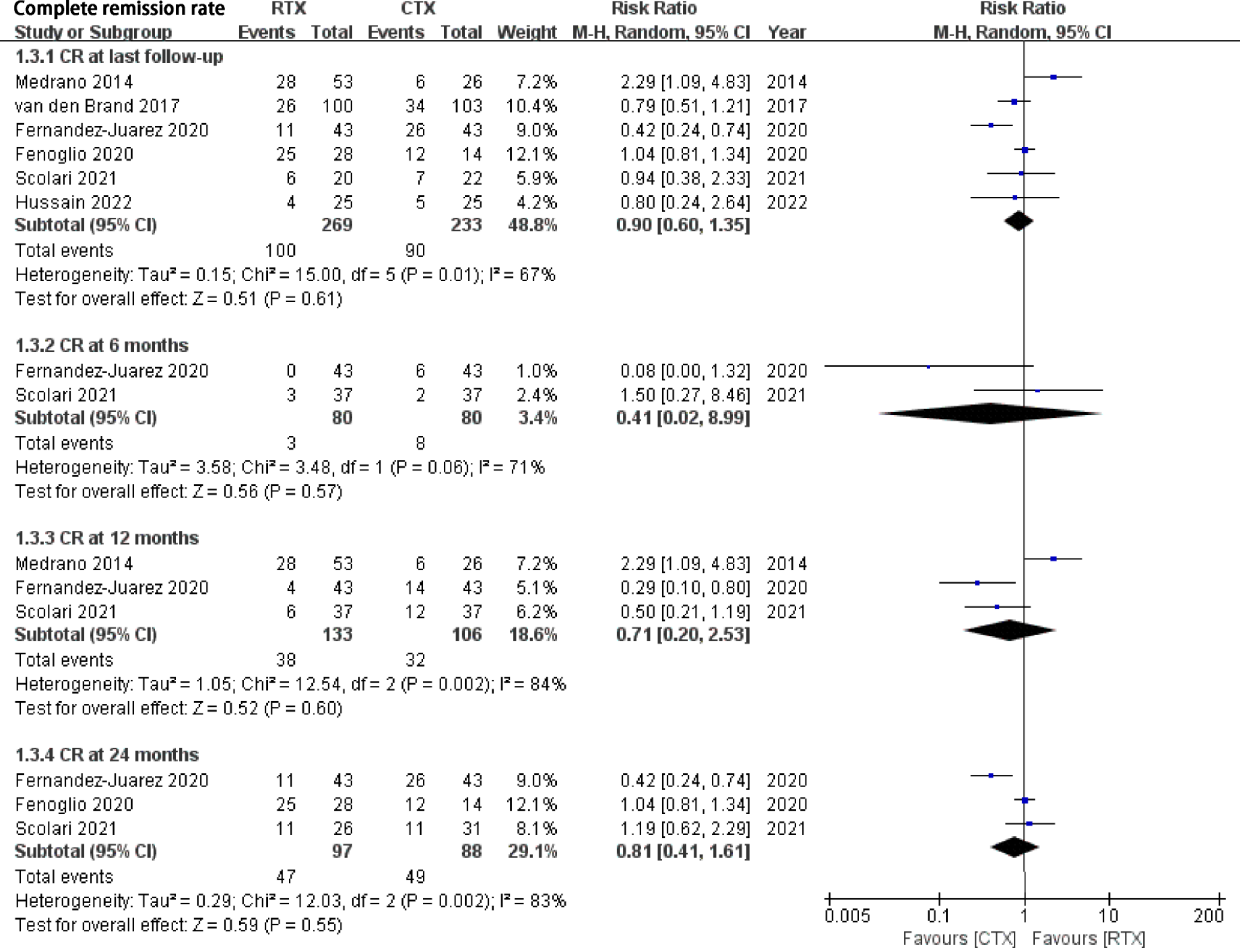
Comparison of complete remission rate between rituximab and cyclophosphamide groups in IMN patients CR, complete remission

### Complete remission rate

RTX treatment was associated with a non-inferior probability of CR rate compared with the CYC group at the last follow-up (6 studies, RR 0.90, 95% CI: 0.60, 1.35, *P* = 0.61, Heterogeneity *I*^*2*^ = 67%, Fig. 3). Publication bias was not significant (Begg’s test: *P* = 0.625, and Egger’s test: *P* = 0.187, Supplement Fig. 4). Sensitivity analysis found stable results. At the follow-up time of 6 months, 12 months, and 24 months, there were no statistical significances of CR rate between RTX and CYC (RR 0.41, 95% CI: 0.02, 8.99, *P* = 0.57; RR 0.71, 95% CI: 0.2, 2.53, *P* = 0.60; RR 0.81, 95% CI: 0.41, 1.61, *P* = 0.55, respectively).

**Fig. 4:**
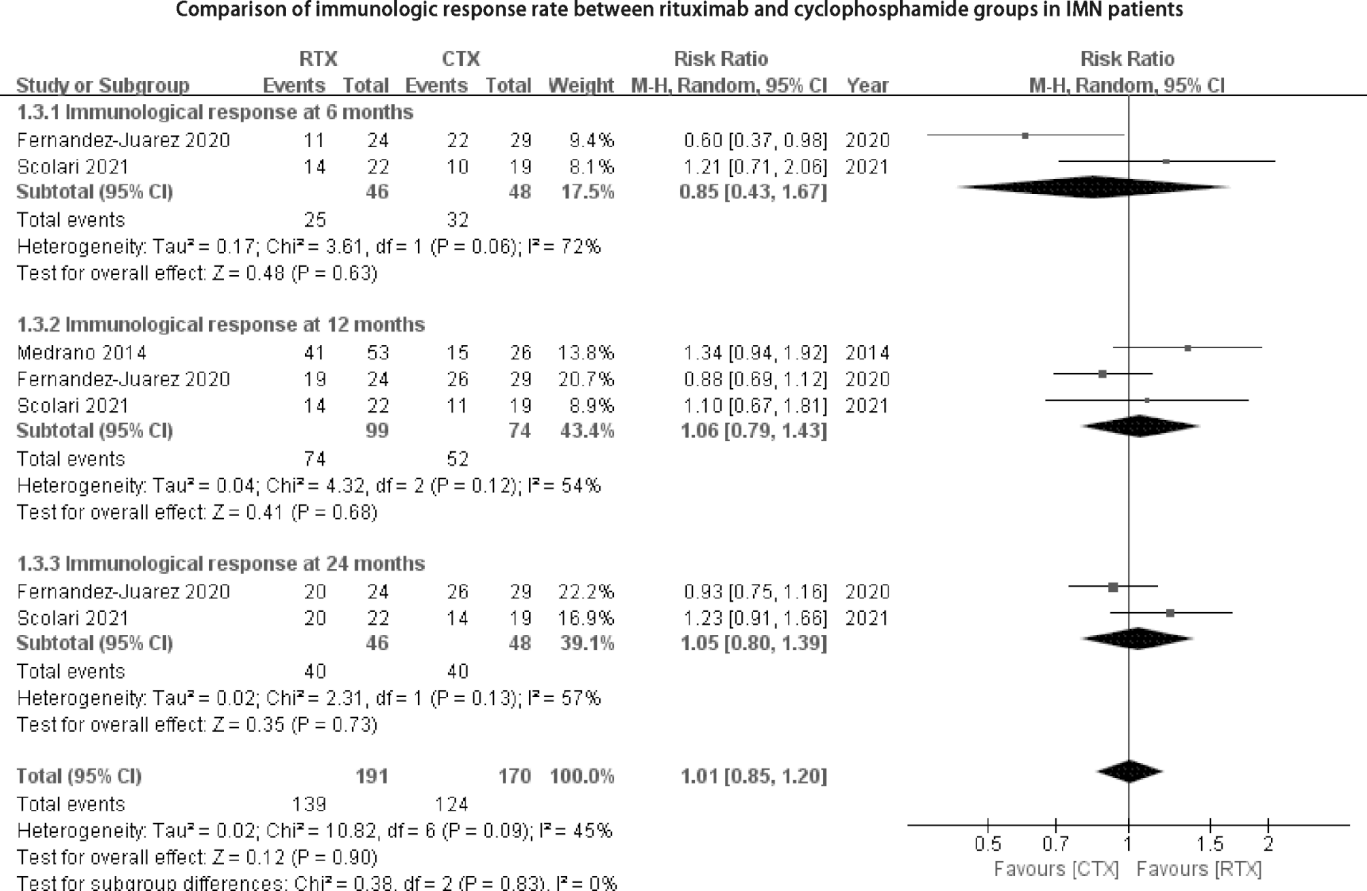
Comparison of immunologic response rate between rituximab and cyclophosphamide groups in IMN patients

### Immunologic response rate

Immunologic response was defined by a level of antiPLA2Rab < 20 RU/ml [14]. At the follow-up time of 6 months, 12 months, and 24 months, there were no statistical significances of immunologic response rate between RTX and CYC (2 studies, RR 0.85, 95% CI: 0.43, 1.67, *P* = 0.63; 3 studies, RR 1.06, 95% CI: 0.79, 1.43, *P* = 0.68; 2 studies, RR 1.05, 95% CI: 0.80, 1.39, *P* = 0.73, respectively, Fig. 4).

### Relapse rate

**Fig. 5:**
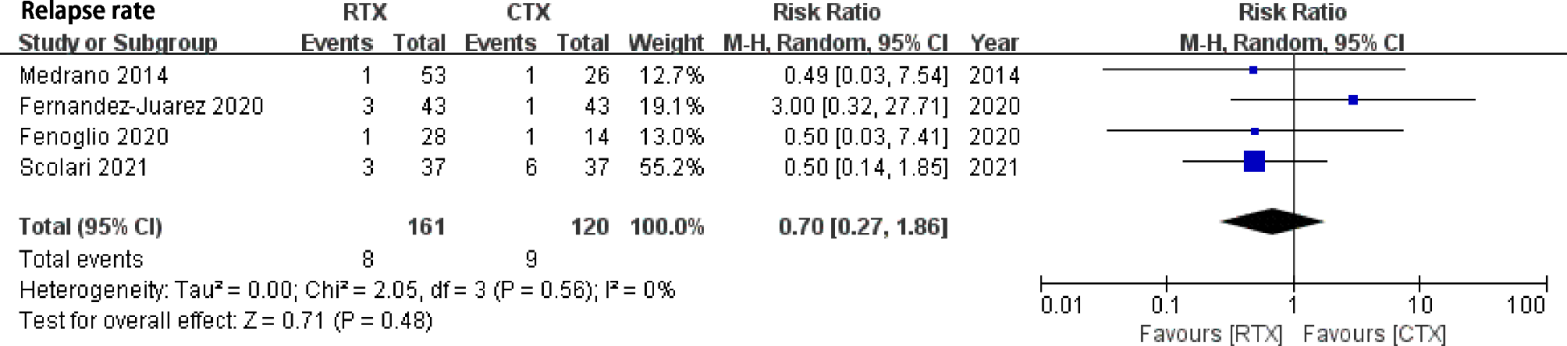
Comparison of relapse rate between rituximab and cyclophosphamide groups in IMN patients

There was no statistically significant difference in the relapse rate between RTX and CYC (4 studies, RR 0.70, 95% CI: 0.27, 1.86, *P* = 0.48, Heterogeneity *I*^*2*^ = 0%, Fig. 5).

### Severe adverse events

**Fig. 6:**
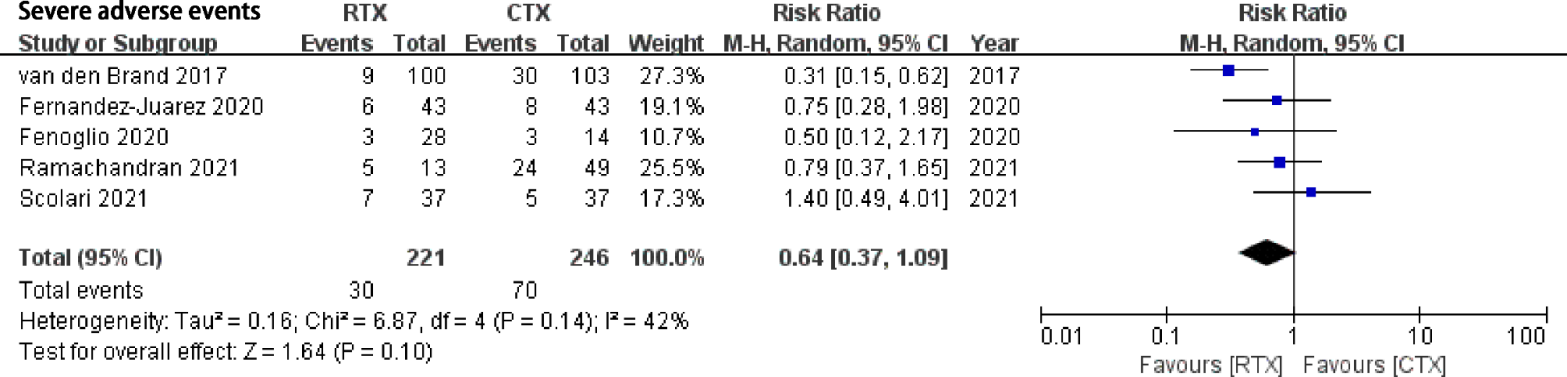
Comparison of rates of severe adverse events between rituximab and cyclophosphamide groups in IMN patients

SAE was defined as death, disability, and a series of life-threatening events. RTX was not associated with a non-significantly lower risk of SAE compared with CYC (Five studies, RR 0.64, 95% CI: 0.37, 1.09, *P* = 0.10, Heterogeneity *I*^*2*^ = 42%, Fig. 6). Publication bias was not statistically significant (Begg’s test: *P* = 0.806, and Egger’s test: *P* = 0.516, Supplement Fig. 5). Sensitivity analysis found stable significant results.

## Discussion

This systemic review compared the efficacy and safety of RTX and CYC treatments for IMN by meta-analysis. We found that although CYC was not significantly different from RTX on risks of long-term proteinuria remission, immunologic response, relapse, and SAE in IMN patients, CYC might be more effective in inducing overall proteinuria remission than RTX in IMN patients with relatively high antiPLA2Rab levels and responded faster in the short term (at 6 months).

Given that the quality of the evidence frequently differed between outcomes, we ranked the quality of the evidence that was most appropriate to each outcome. An overall GRADE quality rating was assigned to a body of evidence across outcomes, typically by selecting the piece of evidence with the lowest quality out of all outcomes that are important for making decisions [21, 22]. Four levels of evidence are used by GRADE: high, moderate, low, and very low [22]. The included studies underwent a thorough quality assessment, utilizing standardized tools such as the Cochrane RoB tool or NOS for observational studies. The overall risk of bias across the two RCTs was the lack of blinding and allocation concealment, highlighting potential limitations [13, 14]. The main risk of bias across the 6 cohort studies came from two small sample-sized retrospective studies with short or inadequate follow-up that might affect the validity of the findings [19, 20]. The GRADE certainty rating of the results in this study was low. However, this study offers valuable insights into the research topic, and its quality assessment supports its credibility and reliability.

According to KDIGO Guideline for glomerular disease in 2021, RTX and CYC are both recommended as the first-line therapy of high-risk IMN patients, which is consistent with our findings [12]. Thus far, the only RCT to directly compare RTX with CYC is the RICYCLO trial. This open-label trial found similar probabilities of CR at 24 months in both groups. Another RCT, the STARMEN trial, indicated that alternating treatment with steroids and CYC was superior to sequential treatment with TAC and RTX in IMN. On one side, the combination of TAC with RTX in an inadequate dose (1 g) would be bound to have a lower remission rate. On the other side, the patients in the TAC/RTX group had higher antiPLA2Rab titers, which might make the RTX less effective.

Three traditional direct meta-analyses have reported the efficacy of RTX or CYC in the treatment of IMN (summarized in Table 3) [23–25]. Zhang et al. found significant differences between RTX and the placebo group in CR rate [24]. Lu et al. found that RTX did not statistically improve the total remission rate (OR = 1.63, 95%CI 0.48–5.54, *P* = 0.43) compared with the control group (including CYC) [25]. Nevertheless, traditional meta-analyses did not distinguish CYC from the immunosuppressive treatments and made the direct comparison between RTX and CYC not clear enough. The recently published network meta-analysis partly overcame this limitation. There have been six network meta-analyses in the treatment of IMN, but none of them found significant differences of remission between RTX and CYC (Table 3) [26–31]. The network meta-analysis by Zheng et al. in 2019 covered 13 immunosuppressive agents in IMN and found that TAC and CYC are superior to other immunosuppressive agents including RTX in terms of total remissions but with no statistical significance [29]. However, two key additional RCTs as STARMEN and RICYCLO have been published since 2019 [13, 14]. The network meta-analysis by Bose et al. in 2022 found that CYC had nonsignificant effects on inducing CR compared with RTX (OR 0.35, CI 0.10–1.24) [31]. Another network meta-analysis by Chen et al. in 2022 showed that RTX and steroid + CYC both were better treatments than others for total remissions in patients with proteinuria < 8 g/d [30]. Compared with the above studies, our direct meta-analysis included more studies and came to novel findings on the efficacy in different follow-up periods and antiPLA2Rab levels.

**Table 3:** Published meta-analysis or systemic reviews about RTX vs. CYC in the treatment of IMN

Author	Year	Meta-analysis type	Number of arms	Number of studies	Treatment group	Control group	Risk ratio of overall remission	Risk ratio of complete remission	Conditions
Lu [25]	2020	Direct meta-analysis	2	8	RTX	Other treatments including CYC	1.6, 95% CI 0.48–5.54	NA	
Zhang [24]	2018	Direct meta-analysis	2	5	RTX	Other treatments including CYC	NA	1.6. 95% CI 0.96–2.66	
Ou [23]	2021	Direct meta-analysis	2	11	RTX	Other treatments including CYC	3.06. 95% CI 1.35–6.94	2.6. 95% CI 0.86–7.89	
Zheng [29]	2019	Network meta-analysis	13	48	CYC	RTX	1.03. 95% CI 0.6–1.7	NA	
Chen [30]	2022	Network meta-analysis	10	25	RTX	CYC	1.32. 95% CI 0.15–11.61	NA	Proteinuria < 8 g/d
					CYC	RTX	1.42. 95% CI 0.21–9.65	NA	Proteinuria > 8 g/d
					CYC	RTX + TAC	1.00. 95% CI 0.15–6.55	NA	Proteinuria > 8 g/d
Bose [31]	2022	Network meta-analysis	13	56	CYC	RTX	NA	0.35. 95% CI 0.10–1.24	
Dai [27]	2020	Network meta-analysis	9	75	RTX	CYC	1.51. 95% CI 0.7–3.21	NA	
Chen [28]	2022	Network meta-analysis	9	24	RTX < 2 g	CYC	0.35. 95% CI 0.08–1.62	NA	At 12 months
					RTX > 2 g	CYC	0.5. 95% CI 0.16–1.57	NA	At 12 months
Liu [26]	2022	Network meta-analysis	12	51	RTX	CYC	1. 95% CI 0.72–1.39	NA	

RTX, rituximab; CYC, cyclophosphamide; NA, not available

The previous meta-analysis did not distinguish the effects of RTX/CYC on patients with different antiPLA2Rab levels. Our study combining direct evidence verified that total remissions in the RTX group were less achievable in patients with relatively high antiPLA2Rab levels > 150 RU/ml. This result was supported by the finding that RTX was less effective in inducing an immunologic remission than CYC in patients with high antiPLA2Rab levels by Van de Logt et al [32]. In patients with the highest tertile of antiPLA2Rab levels (> 150 RU/ml), antiPLA2Rab levels decreased to levels < 14 RU/ml (cutoff value of positive and negative) in 86% of patients treated with CYC, and in 23% of patients treated with RTX [32]. One potential explanation for the differential response is the high burden of antiPLA2Rab in patients with high antibody levels. RTX primarily targets CD20-expressing B cells, while its efficacy against pre-existing antibodies may be limited [33]. In contrast, CYC is an alkylating agent that suppresses immune function by interfering with DNA replication and cellular division. CYC affects a broader range of immune cells, including T cells B cells, and plasma cells, potentially leading to a more robust reduction in antiPLA2Rab levels [34]. The pharmacokinetic and pharmacodynamic properties of RTX and CYC may also play a role in the observed differences. RTX has a longer half-life, allowing for sustained B-cell depletion over time [35]. The sustained B-cell depletion achieved by RTX might be more effective in reducing subsequent autoantibody production in patients with low antiPLA2Rab levels but less efficient in patients with preexisting high antibody titers. Interestingly, in a recent study using a cutoff of 150 RU/ml, antiPLA2Rab levels could identify IMN patients at high risk with a specificity of 80% [36]. Moreover, IMN is a heterogeneous disease, and factors beyond antiPLA2Rab levels may influence treatment response. Variations in underlying immunologic and genetic factors among patients may contribute to differences in treatment outcomes. Further research is needed to better understand the specific characteristics of patients with high antiPLA2Rab levels and their response to different therapeutic approaches.

Another interesting finding of the results was that RTX was associated with a lower CR + PR rate compared with the CYC group at the 6-month follow-up, while there were no significant differences at 12 months and the last follow-up. The differential response time may be attributed to different pharmacokinetics between RTX and CYC. RTX might take a longer time to lower the high antiPLA2Rab levels than CYC. It is postulated that the slower initial response observed with RTX at 6 months could be due to the time required for B-cell repopulation and subsequent immune reconstitution [35]. In contrast, CYC’s broader immunosuppressive effects may lead to an earlier and more rapid reduction in disease activity, resulting in higher response rates at this specific time point. The absence of significant differences in response rates between RTX and CYC at 12 months and the last follow-up suggested that the delayed response observed with RTX at 6 months did not persist over time. Long-term follow-up studies have indicated that RTX may have sustained effects, with response rates eventually reaching similar levels to those achieved with CYC. These findings might help us choose the individualized therapeutic strategy. If an IMN patient with a high antiPLA2Rab titer showed mild and moderate symptoms with no severe complications (such as refractory dropsy, embolism, AKI, and so on), either RTX or CYC plus steroid could be selected. However, when the patient with a high antiPLA2R1ab titer and severe complications was awaiting prompt remission, CYC plus steroid might be a better option. If the patient has some contraindications of steroids, RTX combination with CNI or CYC might be chosen as a candidate strategy.

Besides the comparable efficacy between RTX and CYC, the safety also was compared in this study. Although the result was insignificant, RTX showed a tendency for fewer SAEs. This point coincided with van den Brand et al. who showed that the rates of SAE, including fatal events, were significantly higher in the CYC group [15]. There were a total of 9 deaths in the CYC group, and five were directly attributed to CYC (infections and malignancies); there were 4 deaths in the RTX group, and none were attributed to RTX. Similarly, the nonserious adverse events were significantly higher in the CYC group (127 events) vs. RTX (52 events). The side effects included infections, myelotoxicity, hyperglycemia, and malignancies. However, the safety conclusion still needs more studies to confirm.

Previous literature has shown that inadequate dosing of RTX may impact the remission outcome in IMN [15]. However, we did not find significant differences between low and standard dosing of RTX vs. CYC on CR + PR rate in IMN. There are not enough studies comparing low doses of RTX and CYC in IMN, which necessitates additional research in the future.

This study has several limitations. The sample size of included studies was limited. Although the publication bias and funnel plot results were insignificant, results of this meta-analysis are non-conclusive because of the small number of studies included. The studies were of variable methodological quality. The effect of confounding factors in the cohort studies was not included. There was also considerable heterogeneity concerning participant characteristics (e.g., baseline proteinuria and kidney function), interventions (e.g., CYC vs. RTX + TAC, CYC vs. RTX 375 mg/m^2^, CYC vs. RTX 2 g), outcome definitions (CR and PR), follow-up periods (12 months–6 years) and definition of high levels of antiPLA2Rab. However, the capacity to explore potential sources of heterogeneity due to these factors was limited by the number of included studies. Moreover, we could not divide IMN patients into moderate, high, and very-high-risk groups to further compare the efficacy of the two drugs due to limited data. Also, peripheral CD 19 count was not available, which would be helpful to better establish therapeutic efficacy given the heterogeneity in Rituximab regimens. Last, one paradox of the results was that the IR rate at 6 months was out of accord with the overall remission rate at 6 months between RTX and CYC. Although immunological remission always happens before clinical remission in antiPLA2R-associated IMN patients, IR was calculated in antiPLA2Rab positive patients while the clinical remission rate was based on all patients. Therefore, when only two studies with different proportions of antiPLA2Rab positive INN patients (77% vs. 66%) were included, the immunological remission and clinical remission might be inconsistent [13, 14]. Another possible explanation may be that the STARMEN study prescribed TAC in the first 6 months before RTX, which may lead to the bias of CYC compared with RTX on IR at 6 months [13]. Therefore, the IR results at 6 months still need more studies to prove.

In conclusion, although the long-term efficacy and safety of CYC compared to RTX were comparable, CYC might respond faster and be more advantageous in IMN patients with high antiPLA2Rab titers. The findings emphasize the need for further research and personalized treatment strategies to optimize the management of IMN patients with high antiPLA2Rab levels. The use of antiCD20 agents should be further explored in IMN patients with different antiPLA2Rabs in the future.

### Electronic supplementary material

Below is the link to the electronic supplementary material.

Supplementary Material 1

Supplementary Material 2

Supplementary Material 3

## Data Availability

All data generated or analyzed during this study are included in this published article and its supplementary information files.

## References

[1] Stanescu HC, Arcos-Burgos M, Medlar A, Bockenhauer D, Kottgen A, Dragomirescu L, Voinescu C, Patel N, Pearce K, Hubank M (2011). Risk HLA-DQA1 and PLA(2)R1 alleles in idiopathic membranous nephropathy. N Engl J Med.

[2] Beck LH, Bonegio RG, Lambeau G, Beck DM, Powell DW, Cummins TD, Klein JB, Salant DJ (2009). M-type phospholipase A2 receptor as target antigen in idiopathic membranous nephropathy. N Engl J Med.

[3] Tomas NM, Beck LH, Meyer-Schwesinger C, Seitz-Polski B, Ma H, Zahner G, Dolla G, Hoxha E, Helmchen U, Dabert-Gay AS (2014). Thrombospondin type-1 domain-containing 7A in idiopathic membranous nephropathy. N Engl J Med.

[4] Hoxha E, Reinhard L, Stahl RAK (2022). Membranous nephropathy: new pathogenic mechanisms and their clinical implications. Nat Rev Nephrol.

[5] Hoxha E, Harendza S, Pinnschmidt H, Panzer U, Stahl RA (2014). M-type phospholipase A2 receptor autoantibodies and renal function in patients with primary membranous nephropathy. Clin J Am Soc Nephrol.

[6] Rojas-Rivera J, Fervenza FC, Ortiz A (2022). Recent clinical trials insights into the treatment of primary Membranous Nephropathy. Drugs.

[7] Caravaca-Fontán F, Fernández-Juárez GM, Floege J, Goumenos D, Kronbichler A, Turkmen K, van Kooten C, Frangou E, Stevens KI, Segelmark M (2022). The management of membranous nephropathy-an update. Nephrol dialysis Transplantation: Official Publication Eur Dialysis Transpl Association - Eur Ren Association.

[8] von Groote TC, Williams G, Au EH, Chen Y, Mathew AT, Hodson EM, Tunnicliffe DJ (2021). Immunosuppressive treatment for primary membranous nephropathy in adults with nephrotic syndrome. Cochrane Database Syst Rev.

[9] Bomback AS, Fervenza FC (2018). Membranous nephropathy: approaches to treatment. Am J Nephrol.

[10] Ruggenenti P, Fervenza FC, Remuzzi G (2017). Treatment of membranous nephropathy: time for a paradigm shift. Nat Rev Nephrol.

[11] Oliva-Damaso N, Bomback AS (2021). Rituximab is preferable to Cyclophosphamide for treatment of Membranous Nephropathy: PRO. Kidney360.

[12] Work G, Kidney Disease: Improving Global Outcomes Glomerular Diseases (2021). KDIGO 2021 Clinical Practice Guideline for the management of glomerular Diseases. Kidney Int.

[13] Fernández-Juárez G, Rojas-Rivera J, Logt AV, Justino J, Sevillano A, Caravaca-Fontán F, Ávila A, Rabasco C, Cabello V, Varela A (2021). The STARMEN trial indicates that alternating treatment with corticosteroids and cyclophosphamide is superior to sequential treatment with tacrolimus and rituximab in primary membranous nephropathy. Kidney Int.

[14] Scolari F, Delbarba E, Santoro D, Gesualdo L, Pani A, Dallera N, Mani LY, Santostefano M, Feriozzi S, Quaglia M (2021). Rituximab or Cyclophosphamide in the treatment of Membranous Nephropathy: the RI-CYCLO randomized trial. J Am Soc Nephrol.

[15] van den Brand J, Ruggenenti P, Chianca A, Hofstra JM, Perna A, Ruggiero B, Wetzels JFM, Remuzzi G (2017). Safety of Rituximab compared with steroids and Cyclophosphamide for Idiopathic Membranous Nephropathy. J Am Soc Nephrol.

[16] Ramachandran R, Prabakaran R, Priya G, Nayak S, Kumar P, Kumar A, Kumar V, Agrawal N, Rathi M, Kohli HS (2022). Immunosuppressive therapy in primary membranous nephropathy with compromised renal function. Nephron.

[17] Fenoglio R, Baldovino S, Sciascia S, De Simone E, Del Vecchio G, Ferro M, Quattrocchio G, Naretto C, Roccatello D (2021). Efficacy of low or standard rituximab-based protocols and comparison to Ponticelli’s regimen in membranous nephropathy. J Nephrol.

[18] Medrano AS, Escalante EJ, Caceres CC, Pamplona IA, Allende MT, Terrades NR, Carmeno NV, Roldan EO, Agudelo KV, Vasquez JJ (2015). Prognostic value of the dynamics of M-type phospholipase A2 receptor antibody titers in patients with idiopathic membranous nephropathy treated with two different immunosuppression regimens. Biomarkers.

[19] Hussain AU, Sarween N (2022). Comparison of outcomes between Rituximab and Cyclophosphamide for primary membranous nephropathy: a single Center experience [Abstract]. J Am Soc Nephrol.

[20] Zhou C, Lin S, Cui L, Zhao C. Comparisons of rituximab versus cyclophosphamide in idiopathic membranous nephropathy with high antiPLA2R antibodies [Abstract ]. Chin Soc Nephrol Abstract 2022(PO-0122).

[21] Guyatt GH, Oxman AD, Kunz R, Atkins D, Brozek J, Vist G, Alderson P, Glasziou P, Falck-Ytter Y, Schunemann HJ (2011). GRADE guidelines: 2. Framing the question and deciding on important outcomes. J Clin Epidemiol.

[22] Guyatt G, Oxman AD, Sultan S, Brozek J, Glasziou P, Alonso-Coello P, Atkins D, Kunz R, Montori V, Jaeschke R (2013). GRADE guidelines: 11. Making an overall rating of confidence in effect estimates for a single outcome and for all outcomes. J Clin Epidemiol.

[23] Ou JY, Chen YW, Li TL, Shan HZ, Cui S, Lai JJ, Xiao Y (2022). Evaluation of efficacy of rituximab for membranous nephropathy: a systematic review and meta-analysis of 11 studies. Nephrologie & Therapeutique.

[24] Zhang J, Bian L, Ma FZ, Jia Y, Lin P (2018). Efficacy and safety of rituximab therapy for membranous nephropathy: a meta-analysis. Eur Rev Med Pharmacol Sci.

[25] Lu W, Gong S, Li J, Luo H, Wang Y (2020). Efficacy and safety of rituximab in the treatment of membranous nephropathy: a systematic review and meta-analysis. Medicine.

[26] Liu J, Li X, Huang T, Xu G (2022). Efficacy and safety of 12 immunosuppressive agents for idiopathic membranous nephropathy in adults: a pairwise and network meta-analysis. Front Pharmacol.

[27] Dai P, Xie W, Yu X, Sun J, Wang S, Kawuki J (2021). Efficacy and cost of different treatment in patients with idiopathic membranous nephropathy: a network meta-analysis and cost-effectiveness analysis. Int Immunopharmacol.

[28] Chen M, Zhang X, Xiong Y, Xu G. Efficacy of low or heavy rituximabbased protocols and comparison with seven regimens in idiopathic membranous nephropathy: a systematic review and network meta-analysis. Int Urol Nephrol 2022. 10.1007/s11255-022-03372-5 36161550

[29] Zheng Q, Yang H, Liu W, Sun W, Zhao Q, Zhang X, Jin H, Sun L (2019). Comparative efficacy of 13 immunosuppressive agents for idiopathic membranous nephropathy in adults with nephrotic syndrome: a systematic review and network meta-analysis. BMJ open.

[30] Chen M, Liu J, Xiong Y, Xu G. Treatment of Idiopathic Membranous Nephropathy for Moderate or Severe Proteinuria: A Systematic Review and Network Meta-Analysis. *International journal of clinical practice* 2022, 2022:4996239. 10.1155/2022/4996239 PMC9159126 35685506

[31] Bose B, Chung EYM, Hong R, Strippoli GFM, Johnson DW, Yang WL, Badve SV, Palmer SC (2022). Immunosuppression therapy for idiopathic membranous nephropathy: systematic review with network meta-analysis. J Nephrol.

[32] van de Logt AE, Dahan K, Rousseau A, van der Molen R, Debiec H, Ronco P, Wetzels J (2018). Immunological remission in PLA2R-antibody-associated membranous nephropathy: cyclophosphamide versus rituximab. Kidney Int.

[33] St Clair EW (2010). Good and bad memories following rituximab therapy. Arthritis Rheum.

[34] Ponticelli C, Altieri P, Scolari F, Passerini P, Roccatello D, Cesana B, Melis P, Valzorio B, Sasdelli M, Pasquali S (1998). A randomized study comparing methylprednisolone plus chlorambucil versus methylprednisolone plus cyclophosphamide in idiopathic membranous nephropathy. J Am Soc Nephrol.

[35] Rojas-Rivera JE, Ortiz A, Fervenza FC (2023). Novel treatments paradigms: Membranous Nephropathy. Kidney Int Rep.

[36] Logt AV, Justino J, Vink CH, van den Brand J, Debiec H, Lambeau G, Wetzels JF (2021). Anti-PLA2R1 antibodies as Prognostic Biomarker in Membranous Nephropathy. Kidney Int Rep.

