# Supplementary material for: Cyclophosphamide induced early remission and was superior to rituximab in idiopathic membranous nephropathy patients with high anti-PLA2R antibody levels
Source: BMC Nephrol. 2023 Sep 22;24:280. doi: 10.1186/s12882-023-03307-x (PMC10517553; doi:10.1186/s12882-023-03307-x)
Supplement: Supplementary file 2 — Supplementary Material 2 [file 12882_2023_3307_MOESM2_ESM.docx]

**Supplement table 1. Full search strategy.**

| Pubmed: |
| --- |
| 1IMN ((((((((((((((((((((((Glomerulonephritis, Membranous[MeSH Terms]) OR |
| Glomerulonephritides, Membranous[Title/Abstract]) OR Membranous |
| Glomerulonephritides[Title/Abstract]) OR Membranous Glomerulonephritis[Title/Abstract]) |
| OR Nephropathy, Membranous[Title/Abstract]) OR Membranous |
| Glomerulopathy[Title/Abstract]) OR Glomerulopathy, Membranous[Title/Abstract]) OR |
| Membranous Nephropathy[Title/Abstract]) OR Extramembranous |
| Glomerulopathy[Title/Abstract]) OR Glomerulopathy, Extramembranous[Title/Abstract]) OR |
| Membranous Glomerulonephropathy[Title/Abstract]) OR Glomerulonephropathy, |
| Membranous[Title/Abstract]) OR Idiopathic Membranous Glomerulonephritis[Title/Abstract]) |
| OR Glomerulonephritides, Idiopathic Membranous[Title/Abstract]) OR Glomerulonephritis, |
| Idiopathic Membranous[Title/Abstract]) OR Idiopathic Membranous |
| Glomerulonephritides[Title/Abstract]) OR Membranous Glomerulonephritides, |
| Idiopathic[Title/Abstract]) OR Membranous Glomerulonephritis, Idiopathic[Title/Abstract]) |
| OR Idiopathic Membranous Nephropathy[Title/Abstract]) OR Membranous Nephropathy, |
| Idiopathic[Title/Abstract]) OR Nephropathy, Idiopathic Membranous[Title/Abstract]) OR |
| Heymann Nephritis[Title/Abstract]) OR Nephritis, Heymann[Title/Abstract] |
| 2 Rituximab (("Rituximab"[Mesh]) ) OR (Rituximab)) OR (Rituximab[Title/Abstract]) |
| 3 3Cyclophosphamide (( "Cyclophosphamide"[Mesh] ) OR (Cyclophosphamide)) OR |
| (Cyclophosphamide[Title/Abstract]) |
| 4 #1 AND #2 AND #3 |
|  |
|  |
| Embase: |
| (‘Membranous Nephropathy’/exp OR ‘Membranous Nephropathy’:ab,ti) AND (‘Rituximab’/exp OR ‘Rituximab’’:ab,ti OR ‘Cyclophosphamide’/exp OR ‘Cyclophosphamide’:ab,ti) AND ‘humans’/exp NOT [medline]/lim |
|  |
|  |
| Cochrane library: |
| ID Search |
| #1 MeSH descriptor: [Membranous Nephropathy] explode all trees |
| #2 Kidney disease OR MN OR Membranous Nephropathy OR Kidney Disease |
| #3 #1 OR #2 |
| #4 MeSH descriptor: [Rituximab] explode all trees |
| #5 Rituximab OR anti-CD20 monoclonal antibody |
| #6 #1 OR #2 |
| #7 MeSH descriptor: [Cyclophosphamide] explode all trees |
| #8 #3 and #6 and #7 |

**Supplement table 2**. Assessment of the quality of the included studies by NOS.

| Studies | Selection | | | |  | Comparability |  | Outcome assessment | | | NOS score |
| --- | --- | --- | --- | --- | --- | --- | --- | --- | --- | --- | --- |
|  | ① | ② | ③ | ④ |  | ⑤ |  | ⑥ | ⑦ | ⑧ |  |
| Medrano 2014 | a | a | b | a |  | a |  | b | b | b | 7 |
| van den Brand 2017 | a | b | b | a |  | b |  | b | a | b | 7 |
| Fenoglio 2020 | a | a | b | a |  | a |  | b | a | b | 8 |
| Hussain 2022 | a | a | b | a |  | b |  | d | a | d | 5 |
| Zhou 2022 | a | a | b | a |  | b |  | d | a | d | 5 |
| Ramachandran 2021 | a | a | b | a |  | b |  | b | a | b | 8 |

① Representativeness of the exposed cohort; ② selection of the non-exposed cohort; ③ ascertainment of exposure; ④ demonstration that outcome of interest was not present at start of study; ⑤ comparability of cohorts on the basis of the design or analysis; ⑥ assessment of outcome; ⑦ was follow-up long enough for outcomes to occur; ⑧ adequacy of follow up of cohorts.

**NEWCASTLE - OTTAWA QUALITY ASSESSMENT SCALE**

**COHORT STUDIES**

Note: A study can be awarded a maximum of one star for each numbered item within the Selection and Outcome categories. A maximum of two stars can be given for Comparability

**Selection**

1) Representativeness of the exposed cohort

a) truly representative of the average _______________ (describe) in the community **🟑**

b) somewhat representative of the average ______________ in the community **🟑**

c) selected group of users eg nurses, volunteers

d) no description of the derivation of the cohort

2) Selection of the non exposed cohort

a) drawn from the same community as the exposed cohort **🟑**

b) drawn from a different source

c) no description of the derivation of the non exposed cohort

3) Ascertainment of exposure

a) secure record (eg surgical records) **🟑**

b) structured interview **🟑**

c) written self report

d) no description

4) Demonstration that outcome of interest was not present at start of study

a) yes **🟑**

b) no

**Comparability**

1) Comparability of cohorts on the basis of the design or analysis

a) study controls for _____________ (select the most important factor) **🟑**

b) study controls for any additional factor **🟑** (This criteria could be modified to indicate specific control for a second important factor.)

**Outcome**

1) Assessment of outcome

a) independent blind assessment **🟑**

b) record linkage **🟑**

c) self report

d) no description

2) Was follow-up long enough for outcomes to occur

a) yes (select an adequate follow up period for outcome of interest) **🟑**

b) no

3) Adequacy of follow up of cohorts

a) complete follow up - all subjects accounted for **🟑**

b) subjects lost to follow up unlikely to introduce bias - small number lost - > ____ % (select an adequate %) follow up, or description provided of those lost) **🟑**

c) follow up rate < ____% (select an adequate %) and no description of those lost

d) no statement.
