# Supplementary material for: Cyclophosphamide induced early remission and was superior to rituximab in idiopathic membranous nephropathy patients with high anti-PLA2R antibody levels
Source: BMC Nephrol. 2023 Sep 22;24:280. doi: 10.1186/s12882-023-03307-x (PMC10517553; doi:10.1186/s12882-023-03307-x)
Supplement: Supplementary file 3 — Supplementary Material 3 [file 12882_2023_3307_MOESM3_ESM.docx]

**Supplement figures**

**Supplement figure 1**. Risk of bias of the randomized controlled trials.


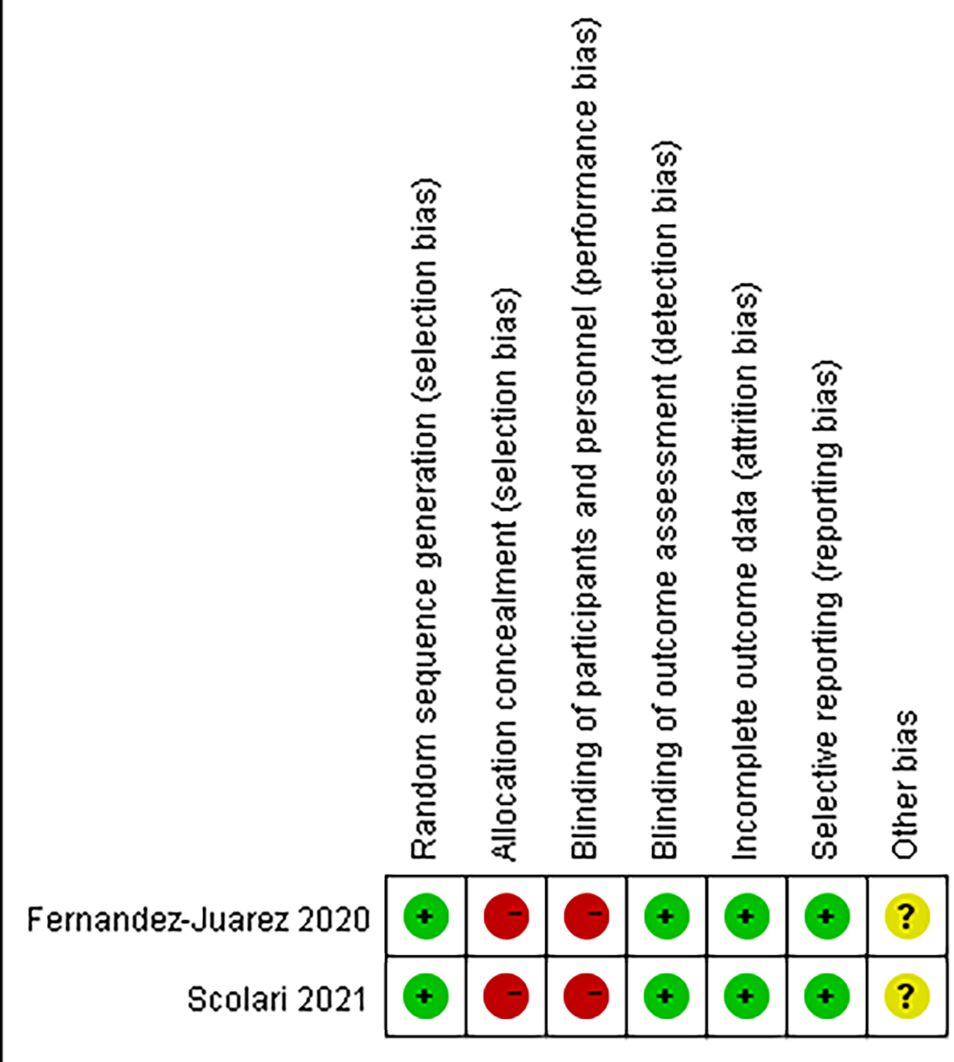


**Supplement figure 2**. Influence analysis and funnel plot of overall complete remission rate.


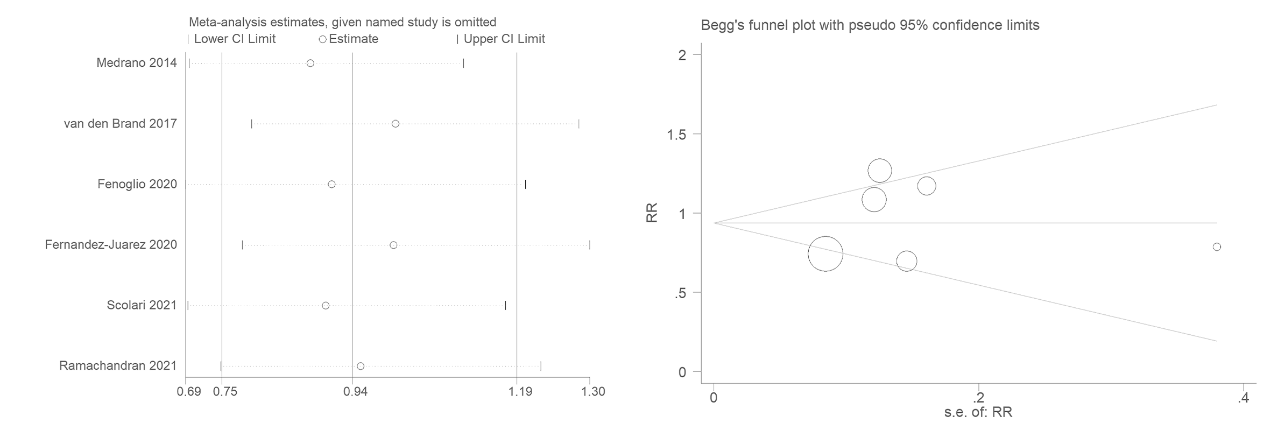


Sensitivity analysis found stable insignificant results.

**Supplement figure 3**. Comparison of complete and partial remission rate between rituximab and cyclophosphamide groups in IMN patients with different clinical settings and treatments.


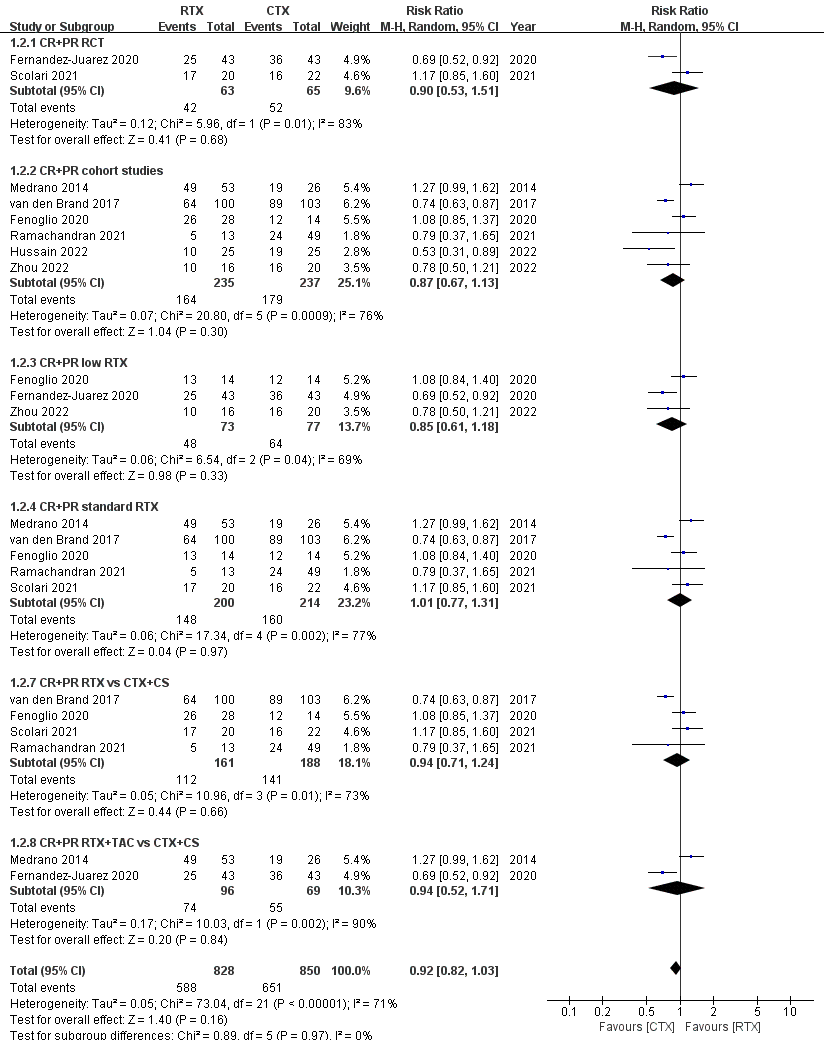


**Supplement figure 4.** Influence analysis and funnel plot of complete remission rate.


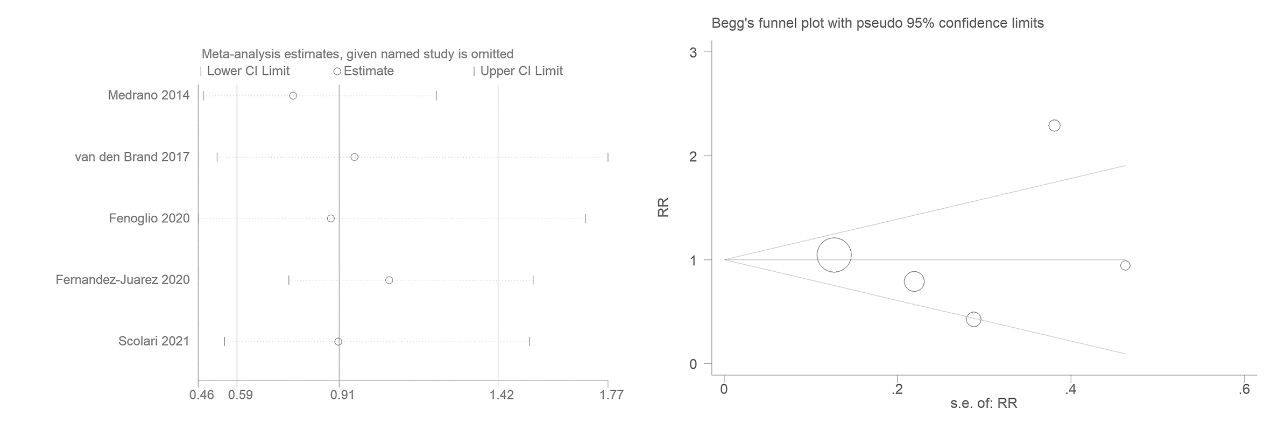


Sensitivity analysis found stable insignificant results.

**Supplement figure 5**. Influence analysis and funnel plot of severe adverse events.


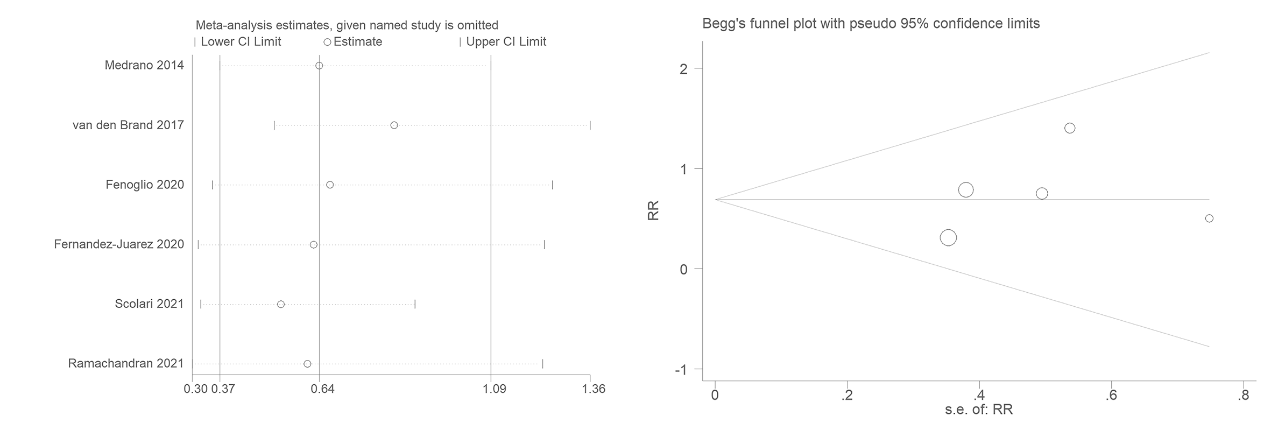


Sensitivity analysis found stable significant results except excluding van den Brand et al.’s study.
